# Boundary Spanning Methodological Approaches for Collaborative Moose Governance in Eeyou Istchee

**DOI:** 10.1007/s00267-023-01918-6

**Published:** 2023-12-25

**Authors:** Nathan A. Badry, Gwyneth A. MacMillan, Eleanor R. Stern, Manuelle Landry-Cuerrier, Gordon M. Hickey, Murray M. Humphries

**Affiliations:** Department of Natural Resource Sciences, McGill University, Montreal, QC Canada

**Keywords:** Knowledge weaving, Indigenous and local knowledge, Co-management, Wildlife, Ontological pluralism

## Abstract

Natural resource governance challenges are often highly complex, particularly in Indigenous contexts. These challenges involve numerous landscape-level interactions, spanning jurisdictional, disciplinary, social, and ecological boundaries. In Eeyou Istchee, the James Bay Cree Territory of northern Quebec, Canada, traditional livelihoods depend on wild food species like moose. However, these species are increasingly being impacted by forestry and other resource development projects. The complex relationships between moose, resource development, and Cree livelihoods can limit shared understandings and the ability of diverse actors to respond to these pressures. Contributing to this complexity are the different knowledge systems held by governance actors who, while not always aligned, have broadly shared species conservation and sustainable development goals. This paper presents fuzzy cognitive mapping (FCM) as a methodological approach used to help elicit and interpret the knowledge of land-users concerning the impacts of forest management on moose habitat in Eeyou Istchee. We explore the difficulties of weaving this knowledge together with the results of moose GPS collar analysis and the knowledges of scientists and government agencies. The ways in which participatory, relational mapping approaches can be applied in practice, and what they offer to pluralistic natural resource governance research more widely, are then addressed.

## Introduction

“We live in a world in which nations govern through science. Indigenous peoples are no exception” (Tallbear 2014, p. 189).

Knowledge pluralism and weaving is an ongoing challenge for natural resource governance researchers and practitioners. There is increasing recognition that natural resources are part of a wider fabric of landscape-level interactions that go beyond disciplinary understandings of social and ecological systems (Reed et al. 2016), and that these interactions involve diverse actors subscribing to diverse knowledge systems (Badry and Hickey 2022). Integrated Landscape Approaches (ILA)—which are “governance strategies that attempt to reconcile multiple and conflicting land-use claims to harmonize the needs of people and the environment and establish more sustainable and equitable multi-functional landscapes” (Reed et al. 2020, p. 1)—are one strategy for addressing the boundaries and complexity of natural resource systems. ILAs attempt to do this by bringing together a wide variety of actors from across spatial, jurisdictional, and knowledge systems boundaries, and finding common ground between them (Reed et al. 2020). Such strategies mark a departure from top-down and state-led understandings of governance, and a movement towards more decentralised understandings, involving multiple levels of government, private-sector actors, and local peoples (DePuy et al. 2021). This departure could be attributed to a variety of drivers, ranging from the broader neo-liberalization of natural resource governance (Turnhout et al. 2014), to the advancement of land claims agreements that enshrine rights to decision-making by Indigenous Peoples (Saku et al. 1998).

While ILAs are explicit about the need to bring together diverse actors, reconciling their multiple and sometimes conflicting knowledge systems and ways of knowing is often a corequisite. This weaving of knowledges is considered a key objective of ILAs, and is thought to increase trust and collaboration (Dressel et al. 2020), as well as enhance evidence-based decision-making through the inclusion of more and different knowledges (Berkes et al. 2000). The inclusion of more and different knowledges, beyond reasons of epistemic justice and representation (Toncheva and Fletcher 2021), means a more complete understanding of the natural world, with the capacity to make better management and policy decisions, and perhaps better achieve shared sustainable-use and conservation goals (Tengö et al. 2017). These knowledges are also not necessarily restricted to the natural world, but can extend to a range of subjects, from environmental ethics to philosophy (Peltier 2018). The Intergovernmental Science-Policy Platform on Biodiversity and Ecosystem Services (IPBES) is one example of institutional support for knowledge weaving. The IPBES has a working group on Indigenous and local knowledge (ILK) systems, with deliverables focussed on “recognizing and working with ILK” within the context of the IPBES and introducing more participatory approaches with knowledge holders (IPBES Secretariat 2019). Within academia, some ecologists have long argued for the inclusion of ILK in applied ecosystem research. They make the case that science and ILK are complementary (Huntington 2000), arguing that different knowledge systems have different strengths and weaknesses, and that by bringing them together, a more holistic approach can emerge. ILK has been noted as being particularly useful for addressing complexity in natural resource systems, and for conducting long-term monitoring (Folke 2004; Bohensky and Maru 2011).

However, in practice, knowledge weaving can be extremely challenging. Obstacles arise because weaving together different knowledge systems requires more than just pooling data, or adapting individual ethical principles (Badry and Hickey 2022). Fikret Berkes, in his work on traditional ecological knowledge, describes knowledge systems as knowledge-practice-belief complexes, which, in addition to specific knowledge of plants and animals, includes management systems, social institutions, and worldviews (Berkes 2018a). Often, during knowledge integration with scientific and bureaucratic knowledges, only the easily integrated elements of ILKs are included (Shaffer et al. 2022).

Knowledge integration is a term commonly used in the applied fields of natural resource management and sustainability. However, it has been argued that “integration” implies a hierarchy of knowledge, in which ILK is only considered reliable if validated by science, and that terms like knowledge co-production, which call for collaboration at every stage of a research project, better describes equitable processes of bringing together different knowledges (Tengö et al. 2014). In this paper, we adopt the term weaving (Popp et al. 2020), and while co-production is crucial, we use weaving specifically to refer to the combining of knowledges, occurring within processes of participation and co-production. We follow Bohensky and Maru by framing weaving “as a process in which the originality and core identity of each individual knowledge system remains valuable in itself, and is not diluted through its combination with other types of knowledge” (2011, p. 10).

During problematic knowledge integration processes, contestations, like whether nonhumans like orcas (Norman 2015) or spirits (Theriault 2017) have agential relationships with people and other nonhumans, and whether these are relevant to governance, can be left out by scientific and bureaucratic knowledge holders. This can lead not only to poor outcomes, but when integrated uncritically, the inclusion of local knowledges might only reinforce existing hierarchies between knowledge systems (Bridel 2022). Other models of bringing together knowledge systems have sought to respond to these criticisms. Two Eyed Seeing is a prominent example. Developed by Mi’kmaq elder Albert Marshall, Two-Eyed Seeing means to see with both eyes: from one eye seeing and learning with Indigenous knowledge, and from the other, seeing and learning with Western knowledge (Bartlett et al. 2012). But while these models provide frameworks and guidance for bringing knowledge systems together, there are still few specific tools for addressing the contestations between them in a way that supports collective action by diverse governance actors (Badry and Hickey 2022).

Contestations between knowledge systems can be framed in ontological terms. Ontology is a branch of philosophy that deals with questions of ‘what is’, and ontological assumptions are assumptions held about what things exist in the world and how they relate to each other. Is an orca a respected, reincarnated chief who should be allowed to remain close to their community, or a wild animal which poses a danger to boat traffic and needs to be relocated (Norman, 2015)? Ontological pluralism describes contexts in which actors hold different ontological assumptions and commitments (DePuy et al. 2021). Sometimes these ontological differences are inconsequential to collaboration. Other times, they can challenge knowledge weaving and collective action, as well as the existing hierarchies between knowledge systems and dominant paradigms of governance. Understandings of environmental “management”, for example, are based on a foundation of ontological commitments often not shared by Indigenous Peoples (Howitt and Suchet‐Pearson 2006). Indigenous Peoples (and many others) may not agree philosophically that aspects of the environment should or can be managed by people, or that the “environment” exists as a meaningful category separate from humans.

Researchers and practitioners in these contexts often apply pluralistic frameworks such as ILAs and Two Eyed Seeing, and are increasingly adapting specific methods to work within them (Reed et al. 2016; Peltier 2018). One such method is Fuzzy Cognitive Mapping (FCM). In this paper, we ask: to what extent can participatory methods like FCM help bridge the ontological gaps inherent to knowledge weaving processes in landscape management, and better support diverse governance actors when they work together towards shared goals? We seek to understand whether FCM represents a meaningful advancement for knowledge weaving and to what extent it can help meet the goals of pluralistic governance actors.

## Fuzzy Cognitive Mapping as a Tool for Knowledge Co-production and Weaving

FCM is a semi-quantitative method of mental modelling, often applied in multi-stakeholder and rightsholder governance contexts. Data for FCM can be collected in a variety of ways, including surveys, interviews, and document analysis (Özesmi and Özesmi 2004). These data are used to identify different nodes, and the relationships, or causal links between them. FCM has been used across a wide variety of fields, including ecology, where the nodes are typically ecological variables (Hobbs et al. 2002; Özesmi and Özesmi 2004). Nodes and relationships are established qualitatively, creating a simplified model of causal relationships (Kosko 1986).

Quantitative weights can then be assigned to each relationship. Adding to the qualitative positive or negative relationship described between each node, a number is assigned, standardized to represent a value ranging from −1 to 1. Much of FCM is rooted in graph theory, similar to other network methods like Social Network Analysis (Poczeta et al. 2019). FCM is a matrix-vector calculation, and measures like eigenvalues and eigenvectors can be used to analyse the adjacency matrices, and give further insights into the roles and importance of different variables (Kok 2009). The fuzzy quantitative values of each relationship can also be worked out collaboratively with participants during data collection, with final values developed through back-and-forth discussion, potentially enhancing the trustworthiness of the approach.

The ‘fuzziness’ in FCM describes the combination of subjective, qualitative knowledge with approximate quantitative values (Alizadeh and Jetter 2017), leading to a fuzzy model. The fuzzy logic of this sort of model potentially makes it more useful for decision-making in systems characterised by complexity and uncertainty (Kok 2009). Fuzzy logic models, similar to ILK systems, allow for more flexible and holistic understandings of the world (Peloquin and Berkes 2009). They deal in large numbers of qualitative variables interrelated by fuzzy relationships. The gaps between different knowledges and approaches can then be bridged by identifying the differences between maps of different knowledge systems subscribers (Hobbs et al. 2002; Sarmiento et al. 2020). FCM can be used descriptively, but, because it is semi-quantitative, it can also be used to develop “what if” scenarios (Voinov et al. 2018). FCM has also been recognised for its usefulness in bringing local and non-academic knowledges into analyses (Gray et al. 2014), including ILK.

However, the effectiveness of FCM for weaving together knowledges in pluralistic natural resource governance contexts, and in particular, for addressing the ontological contestations between knowledge systems, is unclear. FCM offers an interesting method for knowledge weaving because it is semi-quantitative. Semi-quantitative methods have previously been used to bridge gaps and communicate knowledge between different actors, such as between stakeholder and modellers (van Vliet et al. 2010). In what follows, we consider how participatory FCM can be used to support knowledge weaving, as well as consider the opportunities and challenges arising from this approach, using the case of a collaborative moose habitat research project in Eeyou Istchee.

## Research Setting

Eeyou Istchee (), in northern Quebec, Canada, is the traditional territory of the Eastern James Bay Cree. Beginning in the 1950s, the Quebec government increasingly planned development of the northern part of the province, culminating in 1971 with the announcement of the James Bay Hydroelectric Complex. This project was a large-scale development project that would greatly alter the landscape, and it faced organised opposition from Indigenous Peoples in the territory, including the Crees. Following extensive negotiations, in 1975, the James Bay and Northern Quebec Agreement (JBNQA) was signed by the Government of Quebec, the James Bay Energy Corporation, the James Bay Development Corporation, Hydro-Québec, the Grand Council of the Crees (of Quebec), the Northern Quebec Inuit Association, and the Government of Canada. This agreement is widely considered to be one of the first modern land claims agreements with Indigenous Peoples in Canada (Saku et al. 1998). Among other things, the JBNQA was meant to enshrine Cree rights and decision-making within natural resource development processes (Cyr et al. 2022). Notably, Section 24 of the JBNQA establishes a wildlife co-management regime where the Province of Quebec and the Cree Nation share responsibilities for wildlife management.

**Fig. 1:**
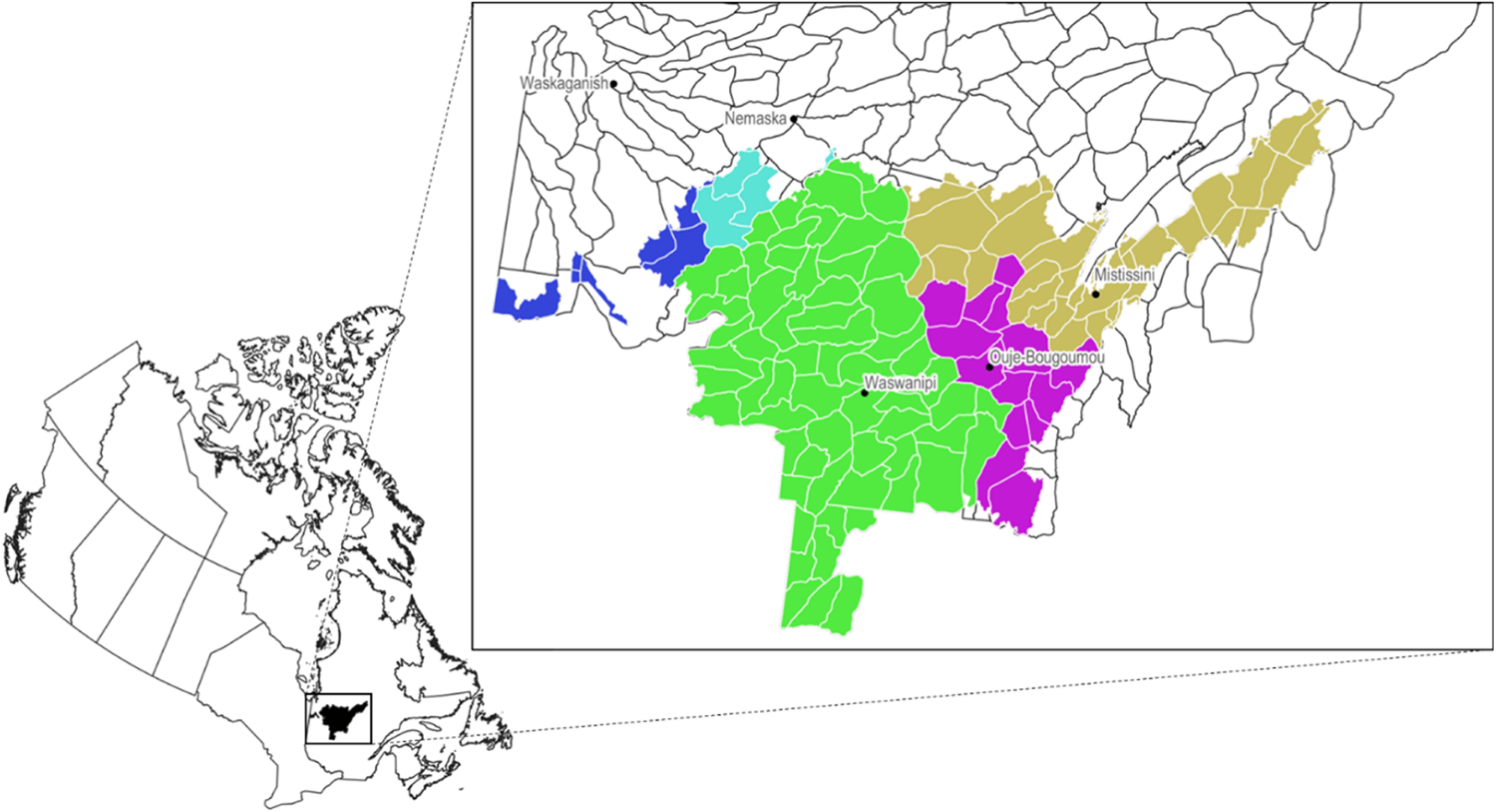
A map of the Adapted Forestry Regime territory, situated within Quebec, Canada, with the individual hunting territories of Mistissini in brown, Nemaska in light blue, Oujé-Bougoumou in purple, Waswanipi in green, and Waskaganish in dark blue (Waskaganish did not participate in the collaborative moose research project)

In 2002, amid concerns by the Crees that the processes and institutions established under the JBNQA did not give them the promised decision-making powers, a new agreement was negotiated. This was the Agreement concerning a new Relationship between the Government of Quebec and the Cree of Quebec, or the “Paix des Braves”. The Paix des Braves was meant to establish a nation-to-nation relationship between the Crees and Quebec, with more collaborative natural resource governance arrangements. In particular, Chapter 3 of the agreement called for an Adapted Forestry Regime (AFR). The AFR covers the southern parts of Eeyou Istchee directly impacted by forestry, and is meant to better account for Cree livelihoods in forestry planning, as well as improve Cree participation and input (Fig. 1). The Paix des Braves also established the Cree-Québec Forestry Board to oversee the AFR, with members nominated by both the Cree Nation Government and the Ministère des Forêts, de la Faune et des Parcs^1^.

The Moose Habitat Quality in Eeyou Istchee Under the Adapted Forestry Regime (MHQ) Project, which began in 2020 and is ongoing, aims to understand and model the various factors influencing moose habitat quality in Eeyou Istchee. The results of this project are considered particularly urgent following 2021 aerial surveys which identified substantial moose population declines in the southern parts of the AFR (Brodeur et al. 2022). The Crees—for whom moose is a wild food species with significant nutritional, economic, social, and cultural value (Bearskin and Berkes 1984)—have expressed strong concerns over these declines. Non-Crees in the territory have also expressed frustrations, as non-Indigenous hunting allowances in some areas were eliminated following the results of the survey (Cree Nation Government 2022). A driving force of the MHQ project is to produce tools that could help guide moose management decisions in the AFR. One of the primary outcomes was a Habitat Suitability Index (HSI) for moose habitat in the AFR, informed by both scientific and Cree knowledges. HSI models are often used in natural resource management to assess the quality of wildlife habitat in a landscape (Brooks 1997). Moose habitat quality and availability is believed by biologists to be a primary driver of moose population dynamics in Eeyou Istchee, and an HSI was especially desired by the provincial government to help guide management and land-use decision-making. Beginning in 2020, a steering committee of local rightsholder and stakeholder organizations was assembled to help guide the MHQ project, including representatives of the Cree Nation Government, the Cree Trappers’ Association, the Ministère des Forêts, de la Faune et des Parcs, the Cree-Québec Forestry Board, and the Cree communities of Mistissini, Nemaska, Oujé-Bougoumou, and Waswanipi, all four of which have territory within the AFR. Fourteen of these steering committee members were interviewed by study co-author Gwyneth MacMillan at the beginning of the project to help understand their different goals and priorities for the research.

## Methods

The MHQ Project offers an instrumental case study (Denzin and Lincoln 2005) of knowledge weaving in natural resource management research in Eeyou Istchee. The MHQ Project is an interdisciplinary and collaborative research effort developing understandings of and modeling the different factors affecting moose habitat. The choice of FCM as a method was driven by the need to bring together Cree and ecological knowledges within a joint project. This need had scientific and technical justifications, but also political ones. The objective of project researchers and steering committee members was that moose collar data, together with Cree expert knowledge, would provide a more complete understanding of the variables affecting moose habitat in Eeyou Istchee, and the effectiveness of the special forestry measures introduced with the AFR:

“It’s going to be important to talk about what [Cree] people know and to understand moose habitat and behaviour from whatever knowledge our people have here. Biologists will have their understanding of all of this, and I know that’s a big part of this project, but it’s going to be important to look at both knowledge types to get a better understanding of the health of the moose population as well as their habitat.”

 - Steering committee representative

Cree knowledge was significant for its potential ecological and biological contributions.However, politically, it was also important that Cree knowledge informed the model, and that tallymen were actively involved in the project, beyond just having Cree representatives on the project steering committee. Cree knowledge and input was a source of legitimacy and a means to exert decision-making power:

“Our Tallyman and our land users are scientists in their own way, you know!”

 - Steering committee representative

**Fig. 2:**
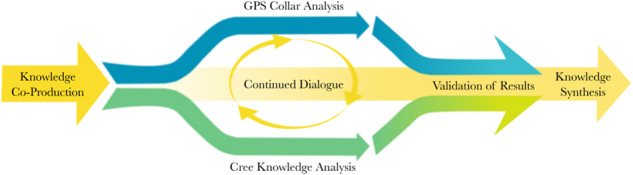
A model of knowledge weaving in the MHQ Project, beginning with a Knowledge Co-Production framework, including project-scoping interviews, and ending with Knowledge Synthesis. Primary streams of data analysis, including analysis of GPS collars and of Cree Knowledge interviews, are represented by blue and green arrows respectively

There were two primary streams of data collection and analysis involved in this project (Fig. 2). One was focussed on the spatial and quantitative analysis of data collected from moose collars, including GPS location and video data. The other focussed on Cree knowledge of moose and moose habitat, and was based mainly on qualitative interviews and semi-quantitative FCM.

We presented our objectives and methods to Chiefs and Councils in each community to inform them of our approach and seek their approval for carrying out our proposed research activities. Band council resolutions in support of the research were obtained in each community prior to data collection, and data collection protocols were reviewed and approved by the McGill University Research Ethics Board (#21-08-034).

### Data Collection and Analysis

#### Habitat selection analysis and moose collar data

Animals have specific habitat requirements related to their need for food, shelter, and safety. Most landscapes consist of a few common habitat types and many rare habitat types. How animals situate themselves within a landscape of contrasting habitat types communicates their individual and species-specific habitat preferences (assuming the animals are free to occupy the habitats most conducive to their own fitness and have the information necessary to make well-informed choices; Fretwell and Lucas 1969). Wildlife habitat selection analyses quantify the habitats used and selected by free-ranging individuals. Habitat use is then often compared to a measure of habitat availability to derive an estimate of habitat selection. Animal habitat use and movement patterns are typically documented by biologists using GPS-equipped biologging devices attached to animals fitted with radio collars. The distribution of animal locations is then compared to the distribution of habitat types. If most animal locations are situated in the common habitats and few in the rare habitats, then use is approximately proportional to availability and habitat selection is considered to be weak or absent. Alternatively, if many, most, or all use locations are clustered in a few uncommon habitat types, then use is disproportionate to availability and these rare but heavily used habitats are considered to be strongly selected or preferred. A simple measure of habitat selection can be obtained by dividing proportional use into proportional availability (Manly et al. 2002).

To develop an HSI for moose within the AFR area of Eeyou Istchee, an understanding of moose habitat selection on the landscape was needed, and this was informed by GPS data and GIS-based land covariate data obtained from the Ministère des Forêts, de la Faune et des Parcs and the Cree Nation Government. Thirty-eight moose collars were employed in the AFR, all affixed to female moose, as female contributions to population productivity were identified as a conservation priority. Moose location data were collected from 2018–2021, recorded every two hours. These data were then used to develop the HSI. Manly selection ratios (proportional habitat use over proportional availability) were calculated for a number of important moose habitat variables, at the scale of the AFR and within each moose’s home range, during both winter and summer (Stern, 2022). These variables were partially informed by key quantifiable environmental drivers to habitat use that were frequently mentioned in interviews with Cree land-users, including land cover, elevation, road density, and distance to water. Winter and summer were identified as highly dichotomous seasons in which differing habitat preferences could be clearly identified. These seasons correspond to the Cree seasons of Niipin, or ‘time for gatherings’ (July to August), and Pipun, or ‘best time to trap’ (January to February; Stern 2022). Home range polygons were created for each moose in winter and summer for each year the collars were on, using Kernel Density analyses to determine home range size and locations.

It should be noted that the collaring of moose began prior to the beginning of the MHQ project and continued throughout, performed by biologists of the Ministère des Forêts, de la Faune et des Parcs. Collaring of animals, and their related capturing and handling, can be controversial, including in Eeyou Istchee and among other Indigenous Peoples (Byers 1999). Concerns often relate to whether the proper respect has been given to the animal, and whether it is safe to consume the animal after it has been tranquilised. Researchers carrying out interviews were informed of the collaring process and health and safety protocols related to the consumption of previously anaesthetised moose.

#### FCM and Cree Knowledge

Members of the research team held 37 interviews with a total of 56 participants in the four communities involved in the project: Mistissini, Nemaska, Oujé-Bougoumou, and Waswanipi (Table 1). We reached out first to the tallymen, or Kaanoowapmaakinch in Cree, in each community. These are the stewards of family hunting territoryin each community, and were contacted due to their depth of knowledge of forestry and moose habitat in Eeyou Istchee. These family hunting territories are called traplines, or Indoh-hoh Istchee, and all of Eeyou Istchee is divided into them. Tallymen are responsible for the land and animals on their trapline, and can exercise control over things like the number of moose that can be harvested during a season. We also contacted land-users, or Indoh-hoh Eeyou, who were known in each community for being particularly knowledgeable about moose. We attempted to organise each interview around a single trapline, with the tallymen and land-users all being from the same hunting territory. However, in some cases this was not possible, with some interviews covering several traplines, and some traplines being split into separate interviews. In communities on the edge of the AFR, traplines affected by forestry were prioritised.

**Table 1:** Number of interviews and participants in each community

Community	Number of interviews	Number of participants
Mistissini	6	8
Nemaska	8	16
Oujé-Bougoumou	8	11
Waswanipi	15	21
Total	37	56

We conducted interviews between October 12^th^ and November 7^th^, 2021. There were still COVID-19 protocols in place in Eeyou Istchee during this time, so the research team worked with local Health and Safety Coordinators in each community to ensure compliance with all public health directives. We organised a one-week quarantine for the research team, followed by COVID-19 screening tests at the hospital, prior to travelling to communities. We also followed masking and sanitation protocols during data collection. Interviews were split between two different teams of researchers which each visited different communities.

**Fig. 3:**
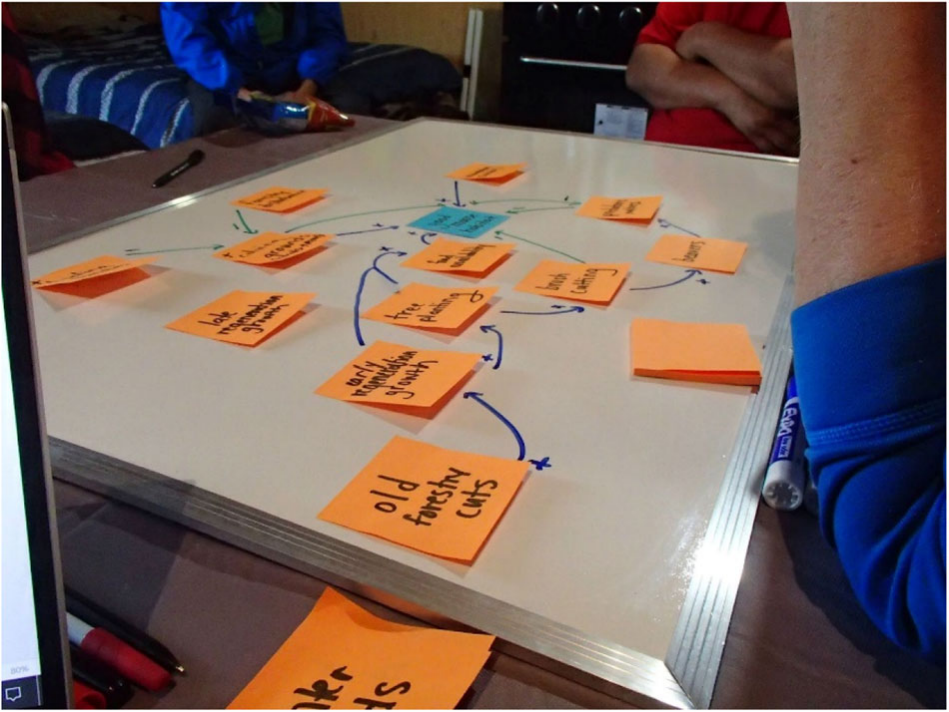
An FCM exercise and interview in process. Variables affecting moose habitat (orange notes) are linked to each other and to the “good moose habitat” (blue note). These linkages are directional, and either positive (blue arrows), or negative (green arrows)

The majority of each interview was dedicated to FCM, although some questions on local forestry and forestry consultations were also asked. FCM data were captured using white boards, markers, and sticky notes. Mapping was done collaboratively with participants during interviews, which lasted for approximately two to three hours (Andersson and Silver 2019). Mapping began with the placing of a central node of “good moose habitat” on a sticky note in the center of the white board. After discussing what “good moose habitat” meant to the participants, the researcher then worked with them to establish the different variables that affect “good moose habitat” on their trapline. These were also written down on sticky notes and placed on the board as nodes. When the participants could not think of any more variables affecting moose habitat on their traplines, the researcher began working with the participants to draw the positive and negative relationships linking these nodes together, and back to “good moose habitat”. Any additional variables that did not arise during the initial discussion, but became evident during the relationship drawing stage, were added at this stage, while existing variables could be reorganised or combined if equivalent. Once all the nodes were linked together by relational arrows, if there was time, the researcher would work with the participants to rank the relative importance of each arrow. The relationships represented by these arrows were ranked from one to five, with a five signifying the most important relationships defining “good moose habitat” on that trapline (Fig. 3).

In each community, research teams worked with local research assistants who helped to reach out to participants, arrange interviews, and translate between Cree and English. Many participants preferred to conduct their interviews in Cree, in which case the research assistant would translate the questions and answers back and forth. Participants received honoraria for participating in the interviews.

Maps were digitised using the software yEd (yWorks). A list of possible variables affecting “good moose habitat” was not established ahead of data collection, allowing the important variables in each interview to emerge inductively in the participants’ own language. However, this necessitated synchronising variables between maps and between research teams. The result was 144 unique variables across 35 maps (two interviews did not result in maps). Fuzzy transitive closure was calculated between each node to account for indirect relationships between nodes (Sarmiento et al. 2020, 2022). Due to the complexity of the data, and to facilitate analysis and communication of the results, thematic analysis was used to group the 144 unique variables into 20 categories (Sarmiento et al. 2020). An adjacency matrix was then developed for the categorised data. Because weights had not been assigned to every map, relationships were weighted according to frequency instead (Sarmiento et al. 2022). Results of participatory relationship weighting, and weighting according to frequency in the data, are typically consistent (Alizadeh and Jetter 2017).

Member-checking workshops were held in each of the four communities in July 2022. Preliminary results from the moose collar and Cree knowledge analyses were presented to previous interview participants and any other interested community members for their impressions and input. The variable categories, in particular, were discussed and validated.

#### Limitations and assumptions

The outcomes of a scientific habitat selection analyses depend on three key analytical decisions: the habitat variables and categories used as a basis for the analysis, the habitats considered to be available to the animal, and the time periods that are included in the analysis. Habitat and landscape classifications, together with other spatial data layers, form the foundation of all habitat analyses. Thus, the reliability of any habitat selection analysis depends on the accuracy, resolution, recency, and relevancy of habitat variables and categories (Fieberg et al. 2021). Commonly deployed GPS satellite collars record precise animal locations (e.g., <15 m of true location) at high fix rates throughout multi-annual sampling periods. Accordingly, habitat use data tends to be highly accurate. Comparatively, characterizing the habitats at each animal’s location is complicated by classification data that can be spatially imprecise, outdated, or incorrect. In our analyses of the GPS collar data, we assume that the classification data are reliable.

The question of which additional habitats are available to an animal—in other words, where the animal is choosing not to be—is an even more complex and scale-dependent issue. Wildlife species, populations, and individuals can be interpreted as selecting habitats at multiple scales, and some of these scales of comparison and choice are more meaningful and relevant than others. A classic contribution to habitat selection analysis classifies three scales of selection as first, second, and third-order selection (Manly et al. 2002), and suggests that analyses of habitat selection should consider available locations across multiple spatial extents to assess multi-scale habitat selection (Fieberg et al. 2021). In our case, the analysis incorporates two scales of selection, at the level of the moose home range, and at the scale of the AFR.

Another key consideration in habitat selection analyses is the temporal extent and seasonal specificity of analyses. If individuals express seasonally divergent patterns of habitat selection, including multiple seasons in a year-round habitat selection analysis may average out and obscure those seasonal differences. We chose to focus on mid-winter and mid-summer in our analysis, as these are time periods where we would expect significant differences in habitat selection. Other seasons have not yet been included in the analysis.

We also made several assumptions during the FCM process. We strove to involve research participants throughout the data collection and analysis process, but the research team still led the process of categorising individual variables. The choice of categories, and how variables were divided among them, was inevitably coloured by researcher positionality and will likely have deviated from how many of our participants would have approached categorization (Sarmiento et al. 2020). To limit this, we focussed on the categorization of variables in our member-checking workshops to ensure that our interpretations still resonated with participants. However, this still posed a challenge for our efforts towards knowledge co-production. When examining the relationships between categories, it also cannot be assumed that every individual variable within those categories reflects those same broad inter-category relationships. Maps of inter-category relationships are aggregated and simplified, and some variability and detail within those categories will have been lost (Johnson et al. 2022). These data and intra-category relationships can still be explored by tracing specific paths within the transitive closure maps and changing the focus and resolution of the map. We also generated qualitive data through the interviews we conducted with participants during the FCM process, and these data were used to guide interpretations of the maps and variables, and reintroduce some of the nuance that could have been lost. A further limitation is that mapping was only conducted with Cree participants. Mapping was not conducted with other potentially interested participants, such as sport hunters, foresters, or government biologists. Therefore, there was no opportunity to contrast the FCMs produced by Cree participants with those produced by the subscribers of different knowledge systems.

Finally, interpretation, and the developing of shared understandings between English and Cree, was sometimes a barrier throughout the interview process. For example, in some interviews, it was difficult to agree on what the concept of “good moose habitat” actually meant, as there was no simple translation to Cree, and the concept is so grounded in scientific knowledge systems. Similarly, later interviews revealed that some participants would assume that when the research team asked questions about moose, they were asking only about male moose. This relates to a broader limitation of this study, in that all the GPS collar data was collected on female moose, but the interviews and FCM included both females and males. The difference could represent an important discrepancy between the two approaches, as female and male moose may behave and select habitat different, for instance, if a cow moose is calving.

## Results

In what follows, we draw on the data collection and analyses conducted through the MHQ project, our own involvement in this project as researchers, and data from available reports and meeting notes. We focus on the process of project development, delivery, and analysis, and conclude with a discussion of future opportunities, challenges, and needs for working across knowledge system boundaries in pluralistic natural resource governance contexts.

### Moose collar data

**Fig. 4:**
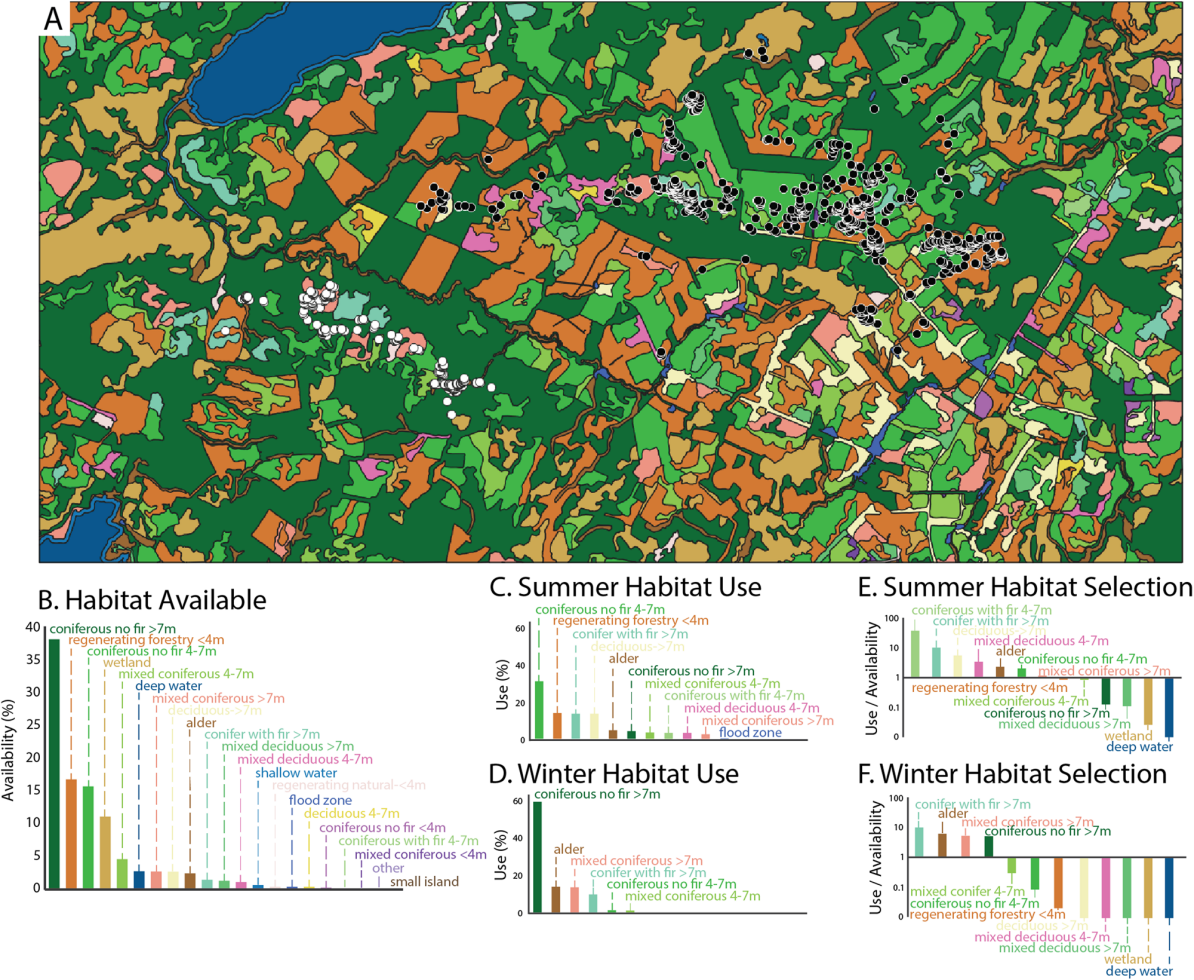
Moose habitat availability, use, and selection in Eeyou Istchee’s Adapted Forestry Regime. **A**. GPS collar locations of a female moose within the Adapted Forestry Regime, including summer (July-August; black points) and winter (January-February; white points) locations, overlaid on coloured polygons reflective of habitat types derived from Quebec’s ecoforestry maps, which include forested areas classified by stand composition and height and additional non-forested habitat types. **B**. Habitat availability, calculated as [(habitat area/total area)x100], with habitat area and total area calculated across the spatial extent included in panel A. **C** Summer habitat use by the representative female moose, based on July-August GPS collar locations. **D**. Winter habitat use by the representative female moose, based on January-February GPS collar locations. **E**. Summer habitat selection, expressed as use relative availability, with availability based on the spatial extent included in panel **A**. **F**. Winter habitat selection, expressed as use relative availability, with availability based on the spatial extent included in panel A

Results of the moose collar data analysis show that moose strongly selected for some habitat types, like mixedwood forests, while avoiding others (Stern, 2022). An example of a habitat selection analysis, simplified from protocols developed by Stern (2022), including consideration of habitats used in winter and summer relative to habitats available, is presented in Fig. 4. This example focuses on a single female moose, whereas the complete habitat selection analysis considered patterns of selection expressed by 38 females across multiple years and scales (Stern, 2022). Further, this example assesses available habitat at a single scale, not corresponding to the scales studied in the full HSI. It uses the spatial extent of a rectangular map as a simple but arbitrary example of how available habitat might be considered, whereas the complete analysis considered two scales of analysis based on random locations distributed within estimated moose home ranges (third order selection) and across the AFR region (second order selection). Accordingly, the results of this simple analysis are illustrative and not necessarily representative of results of the full analysis.

In this example, the predominate available habitats are tall (>7 m) coniferous stands without fir present (38% of total area), regenerating stands following forestry disturbance (16%), medium height (4–7 m) coniferous stands without fir present (16%), and wetlands (10%). These four habitat types collectively account for 80% of land area, whereas 17 different habitat types make up the remaining 20% of land area. Like many other landscapes, this area is composed of a few common habitat types and many rare habitat types. The habitats used by this female moose—determined by overlaying GPS collar locations on habitat categories classified and mapped by the Ministère des Forêts, de la Faune et des Parcs du Québec—indicate use concentrated in a few habitat types and non-use across many habitats in both seasons, although a wider variety of habitat types were used in summer (11 habitat categories; Fig. 4C) than in winter (6 habitat categories; Fig. 4D).

In this single-moose example, combining metrics of use and availability into a selection ratio indicates strong summer preference for tall and medium conifer stands with fir, tall deciduous stands, medium-height mixed stands, and alder stands, combined with strong summer avoidance of deep water, wetland, tall mixed deciduous stands, and tall coniferous stands without fir present, which is the most widely available habitat. Winter selection ratios for this moose indicate a strong selection of alder stands and three tall forest types (mixed coniferous and coniferous with and without fir present) and complete avoidance of deep water (covered by ice in winter), wetlands, tall deciduous stands, and two medium-height stand types.

As stated above, these illustrative results reflect the locations of only one female moose, for a restricted set of predictor variables focused primarily on a forest-focused landcover classification, and do not represent the full habitat selection found in the HSI. A more complete assessment of female moose habitat selection within the AFR, including results from 38 other collared females, two spatial scales of analysis, and additional predictor variables is presented separately (Stern 2022). The example presented here should not be interpreted as results or conclusions about moose habitat selection in the AFR, but rather as an example of the logics and analytical steps involved in the process of habitat selection analyses, and a demonstration of how animals situating themselves within a landscape of contrasting habitat types can help to communicate their individual and species-specific habitat preferences.

### Cree knowledge interviews

The results of the FCM show the relative influence of each habitat variable category (Table 2). Five of the most influential categories on “good moose habitat” were “Hunting and Predation”, “Habitat Features”, “Forestry & Access”, “Noise & Disturbance”, and “Moose Forage”. Each category comprised social and ecological variables. “Hunting & Predation” included variables such as hunting pressure, Indigenous and non-Indigenous hunters on the landscape, poaching, safety while hunting, and nonhuman predators like wolves. “Habitat Features” includes the various habitats that moose use at different times of year, like moose yards, which are moose wintering areas, “typically described as elevated terrain, intersected by valleys, with mature mixed or deciduous stands used for food and mature coniferous stands for cover” (Jacqmain et al. 2005, p. 153). The “Forestry & Access” category included forestry activities more widely, as well as different kinds of cuts and related impacts like debris and increased road access. “Noise and Disturbance” encompassed both natural disturbances like forest fires and windthrow, as well as human disturbances like camps and noisy snowmobiles. “Moose Forage” included a variety of plants that moose feed on seasonally, as well as other broader variables like regrowth and aquatic plants.

**Table 2:** Categories of variables identified affecting moose habitat in Eeyou Istchee and applied in our analyses, not including the central node of “good moose habitat”

Climate & Weather	Habitat Features	Moose Population
Cree Culture	Human Health	Noise & Disturbance
Education & Knowledge	Hunting & Predation	Other Resource Development
Forest Fire	Land Stewardship	Other Wildlife
Forest Management	Moose Forage	Pollution
Forestry & Access	Moose Movement	Protected Areas

The most influential categories varied somewhat between communities. In Oujé-Bougoumou and Waswanipi, the impacts of “Forestry & Access” and “Forest Management” on moose habitat were more clearly reflected in the maps. This is unsurprising, as these communities are further south and have more traplines affected by forestry. Forestry and forestry management was much less relevant in Nemaska, for example, which is at the northern limit of the AFR and has fewer traplines affected by forestry. In Nemaska maps, “Cree Culture” and “Education & Knowledge” had higher relative influence. The “Cree Culture” category includes values like respect and reciprocity, as well as practices like sharing wild foods, preparing moose hides, and practicing the “Cree way of life”. “Education & Knowledge” comprised variables related to knowledge of moose and being on the land, as well the elders and family who passed these teachings on. It should be emphasized that these are measures of relative influence on moose habitat, and not a statement on a category’s overall importance to any community. A more complete assessment of the FCM analysis is presented separately (MacMillan et al. 2023).

### Knowledge Weaving in FCM

FCM as a method helped to illustrate some of the differences between these knowledge systems. By taking an inductive approach in the participatory mapping, and placing nodes in the participants’ own words, we were able to identify a number of new variables affecting moose habitat. The interviews emphasized variables that did not or were unable to emerge from the analysis of habitat suitability. Namely, “Hunting & Predation” and “Other Resource Development”^2^ played important roles in the FCMs, in ways which were not captured in the habitat suitability analysis. “Cree Culture”, Education & Knowledge”, “Human Health”, and “Forest Management” played smaller but still notable roles in many of the FCMs, and similarly were not captured in the moose collar data analysis.

**Fig. 5:**
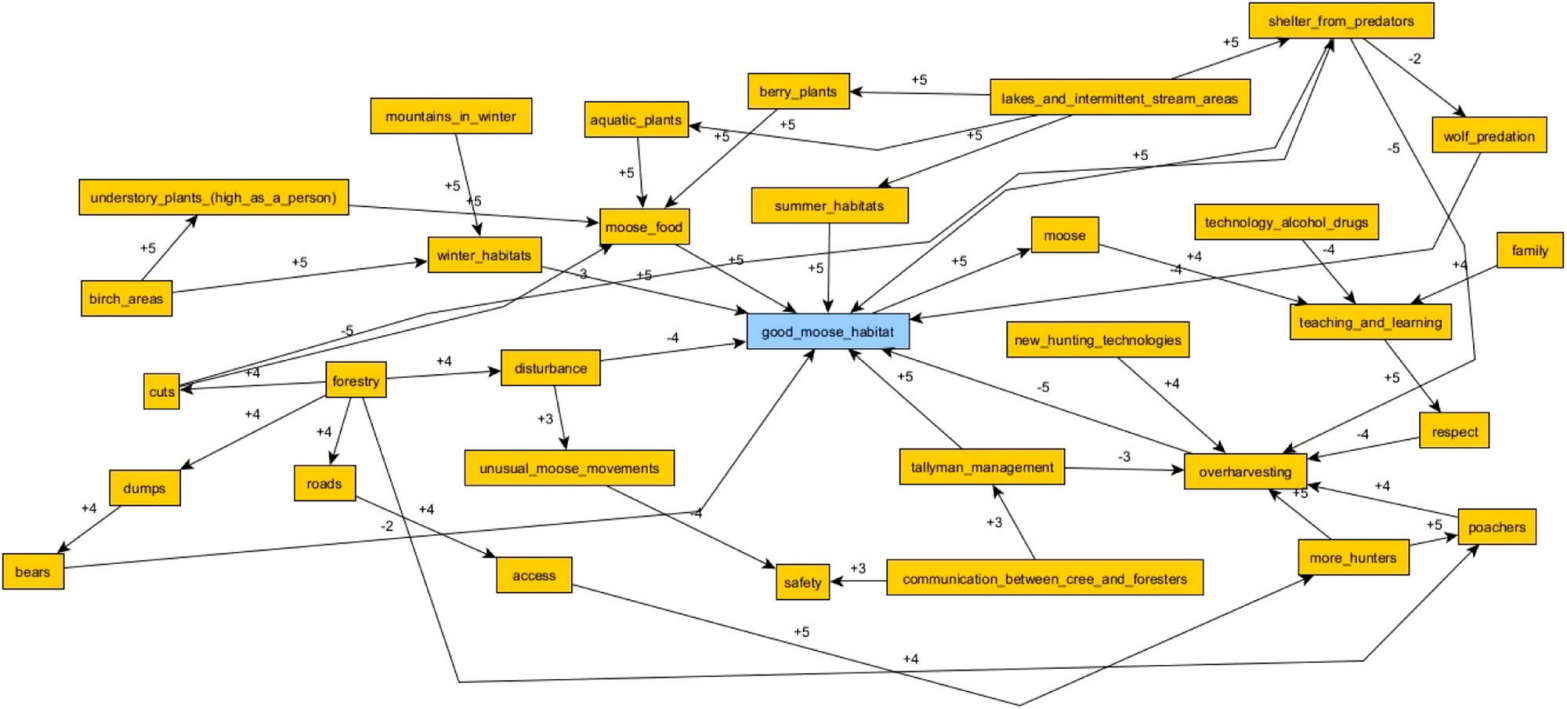
An initial FCM, prior to categorization, from a Mistissini participant, including nonhuman actors like technologies, alcohol, and drugs, and teachings and learning, as well as human actors like family members

Outside of these categories, specific variables like values of respect towards moose, other wildlife, and the land; the importance of familial and inter-generational relationships for transmitting knowledge of moose; and even the effects of social media, drugs, and alcohol on youth, were all raised in mapping exercises (Fig. 5). This provided qualitive insights into how Cree tallymen and land-users understood the relational networks governing moose on their traplines.

The FCMs also showed how important these new variables were quantitatively, either from ranking them with participants during data collection, or through relative frequency after aggregation. The results allowed us to, as Kok describes it, bridge the gap between narrative storytelling and mathematical scenarios (2009). The maps included holistic Cree knowledge of moose habitat, and the importance of many relationships between variables, while also communicating the importance of certain variables in ways that resonated with many of our non-Cree steering committee members. The fuzzy quantitativeness of the relationships allowed for more complex comparisons, like, for example, comparing the relative impacts of poaching between different communities. Poaching is commonly used in Eeyou Istchee to refer to any hunting practices, by Crees or non-Crees, which do not align with traditional Cree hunting law, or Eeyou Indoh-hoh Weeshou-Wehwun.

For the Crees, the identified variables, like respect and knowledge sharing, are critically important for moose habitat. They shape Cree and non-Cree relationships on traplines. Disrespect towards moose and “hunting the wrong way” could lead to excess moose mortality or disturb moose and drive them from the area. This “hunting the wrong way” included poaching and overharvesting, which participants often described as negatively affecting their traplines. Qualities like safety, quiet, and shelter, were very important to Cree understandings of “good moose habitat”. Disturbances come not just from resource development like forestry, but also from more people, both Cree and non-Cree, not practicing land-based activities respectfully. This was in contrast to some of our steering committee members, and particularly non-Cree members, for whom the concept of moose habitat did not necessarily include things like hunters, or quiet, or respect.

**Fig. 6:**
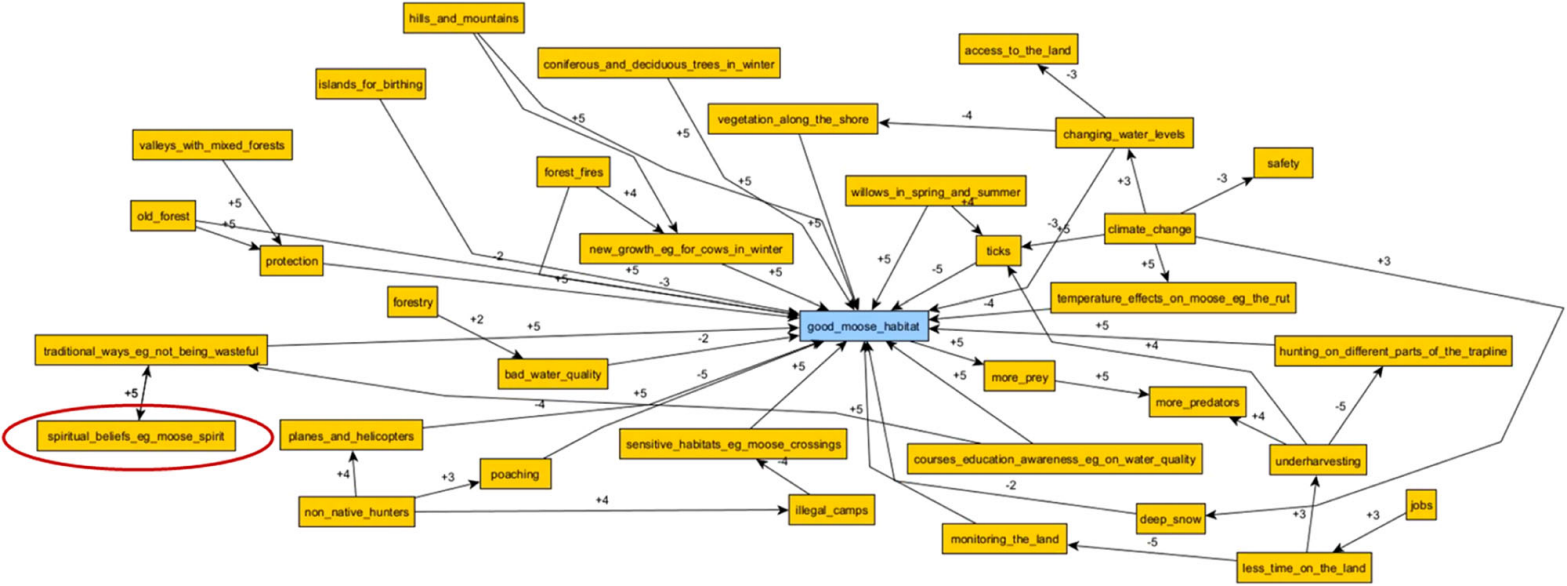
An initial FCM, prior to categorization, from a Nemaska participant which included moose spirit (circled)

However, the role these variables will play in project outcomes is still being determined. Much of the moose GPS data and Cree knowledge data is incommensurable, making any integration of FCM data into the HSI extremely difficult. Spatial and temporal specificity vary considerably between the data sets. The GPS location and habitat data were much more specific than the data from Cree knowledge interviews, which generally applied to the individual participants trapline at its most specific. The complex interactions between FCM variables could be further drawn out, but may appear to some researchers and steering committee members as being outside the boundaries of the research question or priority at hand. Conversely, not drawing them out risks ignoring large elements of expressed knowledge, and missing key connections and relationships. Engagement continues with members of the project steering committee to determine what the HSI should ultimately include to help address positionality biases, as well as what other outcomes should emerge from the project. Nevertheless, it remains likely that due to this incommensurability, some variables like “moose spirit” will remain underrepresented in project outcomes (Fig. 6).

## Discussion

Within the MHQ project, the ‘messy’ work of knowledge weaving is ongoing. Although the HSI model is still in development in collaboration with the project steering committee, the process has progressed far enough to provide some insights for knowledge weaving. In practice, each approach—Moose collars and Cree knowledge—generated data that were unique to the other but also often overlapped. Data from the moose collars provided precise GPS location data, allowing for insights into moose movement and habitat use. Cree knowledge interviews provided more qualitative data on preferred moose food species and moose behaviours related to specific habitats and seasons. These data on food preference and behaviours are further nuanced by video from the moose collars, which show these activities first-hand, albeit mediated by the cameras. One example is moose winter habitat. Analysis of the GPS collar data showed that moose strongly selected for mixed woods forests in winter, and typically moved around less than in summer (Stern 2022). These analyses largely aligned with the analysis of the FCM and interviews, which indicated that winter habitats, which the Cree call moose yards, were typically characterised by a diversity of tree species that moose feed on, like alder and birch, and that these were often areas on hills and mountains which could provide shelter from predators and the elements. These layers of overlapping data have enhanced the trustworthiness of the conclusions drawn from the two approaches (Lincoln and Guba 1985).

The increased trustworthiness of results, and introduction of new variables and directions, supports the claim that FCM is an effective and practical tool for bringing together scientific and ILK systems (Hobbs et al. 2002; Sarmiento et al. 2020). During data collection, the method was flexible. It went at the participants’ own pace, and because it was done physically on a white board, the participant could monitor interview progress, and make corrections and adjustments if they felt something was misrepresented. The method also allowed participants, if they desired, to take more control over the interview, and tell researchers what was important, challenging somewhat the power imbalance between researcher and participant. The data collection dynamic felt well-suited to research in Indigenous contexts, where there can often be distrust towards researchers, and a perception that researchers are just there to ask their questions, extract their data, and leave (Castleden et al. 2012). The participatory nature and duration of the method provided room for rapport and trust to develop.

However, the moose collar and Cree knowledge approaches did not always align. Understanding moose habitat selection related to specific management strategies—like mosaic cutting and the Cree Sites of Special Wildlife Interest, or “25% areas”—was a priority for many of our project steering committee members, who wanted to understand whether these special AFR forestry measures had been effective for preserving and creating moose habitat. 25% areas are the parts of a trapline, adding up to a quarter of the total productive forestry area, that a tallyman can earmark as important wildlife habitat. In these areas there are unique requirements the forestry industry must follow, namely conserving higher proportions of mature forest and leaving more time for regeneration between cuts. Theoretically, these forestry measures would create diversified habitat with both mature and regenerating patches of forest, providing food and cover to moose (Jacqmain et al. 2012). The MHQ Project’s HSI analysis found similar results, showing that moose spent more time in the 25% areas than in habitats outside the 25% areas (Stern 2022). This could be due to the success of the 25% areas, or potentially to other factors, such as tallymen having originally selected the 25% areas because they were naturally better moose habitat than the surrounding trapline. Nevertheless, in our interviews, tallymen and land users often felt strongly that the 25% areas had been ineffective at preserving and creating moose habitat:

“The 25%? It’s just another fancy word.”

 - Waswanipi research participant

Some participants were skeptical that the special forestry measures were effective enough to offset the impacts of development and disturbance on the traplines. Many participants also expressed dissatisfaction with the AFR consultation process. In particular, some tallymen had believed that the trapline areas they had initially chosen to protect wildlife habitat would be free of forestry altogether, rather than subject to a different kind of forestry. The Cree Nation Government and the Government of Quebec, responding to these and other concerns, have been working with tallymen to relocate their 25% areas and improve communication.

According to the accepted forestry and wildlife science informing forest management in Eeyou Istchee, AFR forestry strategies are a key component in the efforts to maintain the proportion of mature and regenerating mixed woods stands, which in turn support moose populations and biodiversity in the territory (Brodeur et al. 2022). Moose use of regrowing stands for food is somewhat supported by our HSI analysis, which found that moose mildly selected regrowing stands post-logging at some scales and in some seasons, although mildly avoided them at others (Stern 2022). For many Cree participants, though, these relationships were more complicated. Some of the complicating variables—like forestry increasing road access and decreasing cover, leading to increased hunting pressure—are also well recognised in the scientific literature (Rempel et al. 1997). Others, like noise disturbance, are not as often acknowledged and would be challenging to quantify for HSI analysis, at least in the holistic sense identified by Cree participants. They identified a range of sources, from land-users, to traffic, to heavy machinery from resource development and construction. Nonetheless, while these two different knowledge systems in this example sometimes reached different conclusions, this is not necessarily a problem or barrier. A lack of agreement is a key element of the knowledge weaving process, and should represent a jumping-off point for further inquiry, rather than an obstacle. But this pluralism can create sticking points when it comes to collaboration and decision-making, as in this example, where the right action to preserve moose populations could vary greatly depending on the knowledge and worldview that is drawn on. Even the project’s focus on moose and moose habitat is grounded in a particular worldview, and has the potential to conflict with Indigenous worldviews, many of which are more holistic (Salomon et al. 2023).

In their work with the Kluane First Nation in the Yukon, Nadasdy refers to knowledge weaving obstacles as “distillation” and “compartmentalization” (1999). When Indigenous knowledge is integrated with science it is often distilled down into its narrowest sense, focussing on specific knowledge of things like plants and animals. By only integrating this most basic level of knowledge, management systems, social institutions, and worldviews are excluded (Berkes 2018a). Things like spiritual values related to moose, or even the role of tallymen on the trapline, are often put aside. But if moose spirit was to be included in an integrated model, how would non-Indigenous decision-makers, or even non-Cree ones, make sense of that? These are ontological obstacles to knowledge weaving. All methods, from interviewing to moose collar analysis, have their own ontological underpinnings and assumptions, and this is an area that requires further exploration in future empirical research. The need was clearly identified by our participants (in this example, speaking in reference to non-Cree hunters):

“If we see their way and they see our way, maybe there could be a better balance”

Oujé-Bougoumou research participant

While FCM is effective at identifying a wide variety of actors, variables, and relationships from across knowledge systems, many of these things may become de-emphasized, de-contextualised, or eliminated completely as maps are aggregated, variables are categorised, and results taken up by decision-makers.

Bringing together science and ILK is a key priority for governance actors in Eeyou Istchee and in many other jurisdictions. However, ontological pluralism and complexity may pose a challenge to knowledge weaving. While some elements of ILK integrate easily with scientific frameworks, like the food species that moose prefer, others, like the role of respect in moose governance, are more challenging to weave together. FCM is a promising method for advancing knowledge weaving in pluralistic contexts like ILAs. It can effectively bring together different knowledge systems to explain complex phenomena. FCM is also semi-quantitative, which facilitates the ability to communicate results to decision-makers, and to incorporate the data into quantitative models. But researchers and practitioners applying FCM are likely to still face challenges in the form of ontological contestations between knowledge systems. We acknowledge too that even if ontological boundaries can be overcome, knowledge weaving in most contexts will still face political challenges (Nadasdy 1999). In Eeyou Istchee, many of the political conflicts have been ongoing since the construction of the James Bay Hydroelectric Project, only now situated in boardrooms and conference calls (Scott 2003).

Within the context of academic research in Eeyou Istchee, this research study builds on several past efforts to bridge gaps between Cree and scientific knowledges. For example, work by Fikret Berkes on Cree knowledge of species like cisco (Berkes 1977) and caribou (Berkes 2018b), which illustrated the value of Cree contributions to ecology and wildlife management. Anthropologist Harvey Feit worked with Cree land-users and tallymen in Waswanipi directly on moose (1987), and demonstrated how Cree practices related to moose were the foundations of traditional management strategies. Hugo Jacqmain et al. complement GPS data from moose collars in Eeyou Istchee with Cree knowledge interviews, contrasting the results between them (2005, 2008). Our research builds on this body of work by assessing the effectiveness of a specific forestry regime, the AFR, which allows Cree tallymen to specify areas within their territories with high wildlife value, and institute specialized forestry practices within these areas intended to maintain wildlife habitats and support Cree ways of life. Additionally, our research is novel in its detailed documentation of two knowledge approaches, one focused on habitat selection analysis of collared moose and the other on FCM of Cree knowledge, conducted within a thorough and explicit knowledge co-production framework. This has allowed us to move towards a model that brings knowledges together in a way in which both inform project results and outcomes equally, without positioning one knowledge above the other. We also sought to be collaborative in our approach, and in doing so brought together a diversity of key governance actors in the territory to have control over the project direction and outcomes, a crucial step, also recognised by Feit, who wrote:

“Native people can no longer use or manage the resources without extensive and effective means of participating in the decisions taken in the wider society which profoundly affect the future of the resources and their use. And government wildlife managers cannot protect or manage the wildlife resources without effective means of participating in the decisions taken in Native society which profoundly affect the future of the wildlife resources and their use” (Feit 1987, p. 40).

Future research on knowledge weaving could focus on assessing the potential of complementary boundary-spanning methods to help navigate some of the obstacles facing researchers working across ontological boundaries. Inspiration could be taken from, for example, recent work on actor-network theory and analytical posthumanism, which may hold insights for addressing boundary-spanning relationships like the ones between “moose spirit” and “good moose habitat” (Badry and Hickey 2022). Such directions have the potential to make the results of transdisciplinary research more responsive to contestations in pluralistic landscapes, better addressing the “landscape of tension” (Parker and Crona 2012) between diverse actors.

## Footnotes

1 In 2022, partway through this research, the Ministère des Forêts, de la Faune et des Parcs was reorganised, with forestry being placed under the purview of the Ministère des Ressources naturelles et des Forêts, and wildlife under the Ministère de l’Environnement, de la Lutte contre les changements climatiques, de la Faune et des Parcs. For clarity we will use the former name throughout.

2 This category did not include forestry, which was categorised separately, but included development activities like mining and hydroelectric development.
